# Improve BBB Penetration and Cytotoxicity of Palbociclib in U87-MG Glioblastoma Cells Delivered by Dual Peptide Functionalized Nanoparticles

**DOI:** 10.3390/pharmaceutics15102429

**Published:** 2023-10-06

**Authors:** Yu-Chen Lo, Wen-Jen Lin

**Affiliations:** 1 School of Pharmacy, College of Medicine, National Taiwan University, Taipei 10050, Taiwan; elisa50626@gmail.com; 2 Drug Research Center, College of Medicine, National Taiwan University, Taipei 10050, Taiwan

**Keywords:** palbociclib, nanoparticles, dual peptide, glioma cells

## Abstract

Palbociclib (PBC) is an FDA-approved CDK4/6 inhibitor used for breast cancer treatment. PBC has been demonstrated its ability to suppress the proliferation of glioma cells by inducing cell cycle arrest. However, the efflux transporters on the blood-brain barrier (BBB) restricts the delivery of PBC to the brain. The nano-delivery strategy with BBB-penetrating and glioma-targeting abilities was designed. Poly(lactide-co-glycolide)-poly(ethylene glycol) (PLGA-PEG) was functionalized with the potential peptide, T7 targeting peptide and/or R9 penetrating peptide, to prepare PBC-loaded nanoparticles (PBC@NPs). The size of PBC@NPs was in the range of 168.4 ± 4.3–185.8 ± 4.4 nm (PDI < 0.2), and the zeta potential ranged from −17.8 ± 1.4 mV to −14.3 ± 1.0 mV dependent of conjugated peptide. The transport of PBC@NPs across the bEnd.3 cell model was in the order of dual-peptide modified NPs > T7-peptide modified NPs > peptide-free NPs > free PBC, indicating facilitated delivery of PBC by NPs, particularly the T7/R9 dual-peptide modified NPs. Moreover, PBC@NPs significantly enhanced U87-MG glioma cell apoptosis by 2.3–6.5 folds relative to PBC, where the dual-peptide modified NPs was the most effective one. In conclusion, the PBC loaded dual-peptide functionalized NPs improved cellular uptake in bEnd.3 cells followed by targeting to U87-MG glioma cells, leading to effective cytotoxicity and promoting cell death.

## 1. Introduction

Glioma is known to be one of the most aggressive and devastating diseases. Due to the heterogeneity, invasiveness, and infiltration of gliomas, patients typically have a median survival time of around one year, and only 5% survive for more than five years. Systemic chemotherapy is considered an essential treatment for gliomas, regardless of whether surgery is available or not [1]. However, the side effects of chemotherapy cannot be ignored, particularly in the residual site within the central nervous system (CNS). Another important issue to consider is the development of resistance during chemotherapy. Shah et al. found that certain phytocompounds can interfere with several oncogenic proteins, inhibiting cancer metastasis, angiogenesis, and therapeutic resistance [2]. Other studies have focused on developing biocompatible hydrogel-based delivery systems that encapsulate chemotherapeutic drugs to improve glioblastoma therapy outcomes [3].

Palbociclib (PBC) is a potent inhibitor of cyclin-dependent kinase 4/6 (CDK4/6), which leads to the inhibition of Rb1 phosphorylation and eventual cell cycle arrest. PBC was approved for the treatment of ER+/HER2- breast cancer in US [4]. The potential use of PBC in other cancer therapies is under wide investigation due to dysregulated cyclin D-CDK4/6-Rb pathway occurring in many types of tumors [5,6]. In vitro studies have demonstrated that PBC induces cell cycle arrest not only in breast cancer but also in glioma cancer cells [7]. Additionally, the Cancer Genome Atlas Research Network (TCGA) revealed that dysregulation of the cyclin D-CDK4/6-Rb pathway was found in 78.9% of glioblastoma cases [8]. As the CDK4/6 pathway is highly dysregulated in glioma, the CDK4/6 inhibitor is considered an attractive novel therapeutic target for the treatment of glioma. Moreover, Barton et al. applied PBC in a preclinical study for brainstem glioma, which exhibited significantly prolonged survival in a mouse model [9]. All these findings support PBC as a potential candidate for glioma therapy. However, two dominant efflux transporters highly expressed at the blood-brain barrier (BBB) limit the delivery of PBC into the brain. Unfortunately, PBC is a substrate of breast cancer resistance protein (BCRP/ABCG2) and p-glycoprotein (P-gp/ABCB1), thereby greatly restricting its delivery into the brain [10,11]. To overcome this hurdle, several strategies are proposed, including pharmacological inhibition of active efflux transporters, refinement of the drug structure to allow passive diffusion through the BBB, or delivery by nanocarriers to evade the efflux transporters [12,13,14].

The T7 peptide (His-Ala-Ile-Tyr-Pro-Arg-His) is a seven amino acid peptide that was identified through phage display systems. Studies have shown that it has a high affinity for the human transferrin receptor (TfR) and can bind to it with a comparable strength as holotransferrin [15]. It is worth noting that the binding site of T7 peptide on TfR is distinct from that of endogenous transferrin, meaning there is no competition for receptor sites, and the homeostasis of iron is still well-regulated [16,17,18,19]. This makes T7 peptide an ideal targeting ligand with specific binding to TfR for anticancer drug delivery. In fact, studies have shown that the expression levels of TfR in glioma cancer cells and brain endothelial cells are higher than in normal cells, making T7 peptide a promising candidate for targeted drug delivery in cancer therapy [20,21,22,23].

Cell penetrating peptides (CPPs) possess the unique ability to traverse the cell membrane and deliver cargoes into cells [24]. CPPs are often conjugated with various cargoes to facilitate the cellular uptake of therapeutic agents via electrostatic interaction with the cell membrane. The degree of adsorption between particles and the cell membrane is dependent on several factors, such as the size, shape, charge of the particles, the number of grafted CPPs, and the conformational flexibility of the CPP-grafted particles [25]. CPPs are used extensively to transport various biologically active cargoes, such as genes, proteins, nucleic acids, siRNA, and so on [26,27,28,29,30]. Recently, a 15-amino acid linear peptide was designed to complex with pDNA as a gene delivery vector. The peptide-DNA cationic nanoparticles demonstrated successful transfection in breast and prostate cancer cell lines and in vivo antitumor efficiency via intravenous delivery [26]. Feldman et al. used phage display to identify a 12-amino acid CPP that targets the heart and allows for rapid uptake after intravenous injection. It was applied to deliver macromolecular therapeutics for acute myocardial infarction [31]. The CPP-macromolecular therapeutics conjugate is another approach not only for cancer therapy but also for trans-BBB targeting, lung targeting, and muscular dystrophies [28,32]. Although arginine-rich CPPs are widely employed, the cytotoxicity of polyarginine peptides is concerned [33]. Some researches provide the strategies to shield the CPPs and minimize their exposure to the normal cells [34].

In this study, we developed a nano-delivery system that can penetrate BBB and target glioma cells for a cyclin-dependent kinase inhibitor, palbociclib. The main material used to prepare nanoparticles (NPs) was poly(lactide-co-glycolide) (PLGA), an FDA-approved polymer known for its safety in clinical use. To enhance targeting, we used a short-chain T7 peptide, which has high affinity to TfR, as the targeting ligand. Additionally, the cationic R9 peptide, known for its prominent cell-penetrating ability, was utilized to facilitate the nanocarriers’ entry into cells via a receptor-independent pathway. The T7 and R9 peptides were planned to conjugate onto the NPs using different chain lengths of poly(ethylene glycol) (PEG). The PBC loaded peptide-modified NPs were prepared and characterized, and the cellular uptake in bEnd.3 cells and the effective cytotoxicity in U87-MG glioma cells were investigated and compared to the peptide-free NPs.

## 2. Materials and Methods

### 2.1. Materials

Poly(D,L-lactide-co-glycolide) 50:50 was from Evonik Industries (Birmingham, AL, USA). 1-(3-Dimethylaminopropyl)-3-ethylcarbodiimide hydrochloride (EDC), N-ethyldiisopropylamine (N,N-Diisopropylethlamine) (DIEA, 99%), and thiazolyl blue tetrazolium bromide (MTT, 98%) were from Alfa Aesar (Heysham, UK). N-hydroxysuccinimide (NHS, 98%) was from Acros Organics Co. Inc. (Fair Lawn, NJ, USA). T7-peptide (FITC-Asp-His-Ala-Ile-Tyr-Pro-Arg-His-OH) and R9-peptide (Arg-Arg-Arg-Arg-Arg-Arg-Arg-Arg-Arg-OH) were synthesized by Kelowna International Scientific Inc. (Taipei, Taiwan). Palbociclib (PBC, 98%) was from Hunan HuaTeng Pharmaceutical Co., Ltd. (Changsha City, Hunan, China). Polystyrene standards were from Sigma-Aldrich Co., Ltd. (St. Louis, MO, USA). Poly(vinyl alcohol) (PVA, 88% hydrolyzed) was from Acros Organics Co. Inc. (Fair Lawn, NJ, USA). Phosphotungstic acid (PTA) was from Electron Microscopy Sciences (Hatfield, PA, USA). bEnd.3 and U87-MG cell lines were from Bioresource Collection and Research Center (Hsinchu, Taiwan). Dulbecco’s Modified Eagle Medium (DMEM) powder was from Thermo Fisher Scientific Inc. (Grand Island, NY, USA).

### 2.2. Synthesis and Characterization of Peptide-Conjugated PLGA-PEG Copolymers

PLGA-PEG was synthesized based on our previously published method [35]. PLGA was pre-activated to PLGA-NHS followed by reaction with different chain lengths of PEG_5k_ or PEG_2k_ via amide linkage. The peptide-conjugated PLGA-PEG_5k_-T7 and PLGA-PEG_2k_-R9 copolymers were then synthesized [36,37]. Specifically, T7-peptide and R9-peptide were reacted with PLGA-PEG_5k_ and PLGA-PEG_2k_, respectively, at a molar ratio of 1:1.5 in the presence of NHS and EDC (molar ratio 1:1) at room temperature for 24 h in the dark. The synthesized copolymers were precipitated with ice-cold diethyl ether and centrifuged at 5000 rpm for 10 min at 4 °C. The resulting mixture was washed with ice-cold diethyl ether/methanol (8/2, *v*/*v*) three times to remove unreacted compounds and then dried in a desiccator under vacuum.

The molecular weight of the peptide-conjugated copolymers was determined by size exclusion chromatography (SEC) using a Styragel HR 4E column (7.8 × 300 mm, Waters, Milford, MA, USA) equipped with a refractive index detector (RI 2031 Plus, Jasco International Co., Ltd., Tokyo, Japan). HPLC-grade chloroform was used as the mobile phase, and the flow rate was set at 1 mL/min at 35 °C. Prior to injection, the copolymers were dissolved in chloroform and filtered through a 0.22-μm nylon syringe filter. The calibration curve was constructed using polystyrene standards, and the weight-average molecular weight (Mw), number-average molecular weight (Mn), and polydispersity (PD) of PLGA-PEG_5k_-T7 and PLGA-PEG_2k_-R9 were calculated based on the calibration curve. The critical micelle concentration (CMC) was determined using pyrene as a fluorescence probe [35,38]. The emission wavelength was set at 390 nm, and the excitation fluorescence was recorded at 336 nm and 333 nm by a fluorescence spectrophotometer (SPECTRA MAX GEMINE XS, Mississauga, ON, Canada). The CMC value was determined from the plot of the fluorescence ratio of I_336_/I_333_ versus logarithmic concentrations of peptide-conjugated copolymers. The concentration at the intersection of the steep and the horizontal parts of the curve corresponded to the CMC value.

### 2.3. Preparation and Characterization of PBC Loaded NPs (PBC@NPs)

The PBC loaded NPs, PBC@NPs, including PBC@PP_5k_/PP_2k_ NPs, PBC@PPT_5k_/PP_2k_ NPs and PBC@PPT_5k_/PPR2k NPs, were prepared using a single solvent evaporation method [35,36,37]. Briefly, the polymer and PBC were dissolved in dichloromethane (1:0.06% *w*/*v*) as the oil phase. PVA (0.5% *w*/*v*) in a sodium bicarbonate buffered solution (0.5 M) served as the water phase. The oil phase was slowly added into the water phase (1:10 *v*/*v*) under sonication in an ice bath, followed by magnetic stirring for 12 h. The residual organic solvent was removed by a rotary evaporator under reduced pressure at 35 °C. PBC@NPs were collected after centrifugation at 70,000× *g* for 30 min at 4 °C. The PBC@NPs were subsequently resuspended in deionized water and centrifuged. The same washing process was repeated twice. Finally, the PBC@NPs were frozen at −80 °C followed by freeze-dried (EZ-550R, FTS Systems, Stone Ridge, NY, USA), and the yields of PBC@NPs were calculated. The morphology of PBC@NPs was observed by transmission electron microscope (TEM) (Hitachi High-Technologies Corporation, Tokyo, Japan). The PBC@NPs suspension was placed on copper electron microscopy grids, PTA solution (2% *w*/*v*) was dropped and stood for 30 s. After removing the excess fluid, the samples were dried at room temperature prior to TEM observation. The particle size and zeta potential were determined by zetasizer (Nano-ZS 90 Zetasizer, Malvern Instruments, Worcestershire, UK). The PBC@NPs were dissolved in the mobile phase containing acetonitrile and sodium dihydrogen phosphate buffered solution (0.01 M, pH 4.5) at 7:3 *v*/*v*, and the concentration of PBC was then determined by HPLC (PU-2089 plus, Jasco International Co., Ltd., Tokyo, Japan) equipped with a C18 column (Luna 5 μm, 4.6 × 250 mm, Phenomenex (Torrance, CA, USA)) and a UV detector (MD-2010 plus, Jasco UV, Jasco International Co., Ltd., Tokyo, Japan) at 357 nm. The flow rate was set at 1.0 mL/min. The calibration curve of PBC was established, and the concentrations of PBC encapsulated by NPs were measured according to the calibration curve. The drug encapsulation efficiency (EE, %) and drug loading (DL, %) were calculated using the following equations.

(1) $$\mathrm{Encapsulation}\;\mathrm{efficiency}\;(\%)=\frac{\mathrm{Determined}\;\mathrm{amount}\;\mathrm{of}\;\mathrm{PBC}\;\mathrm{in}\;\mathrm{NPs}\;}{\mathrm{Amount}\;\mathrm{of}\;\mathrm{PBC}\;\mathrm{added}\;\mathrm{initially}}\times 100\%.$$

(2) $$\mathrm{Drug}\;\mathrm{loading}\;(\%)=\frac{\mathrm{Determined}\;\mathrm{amount}\;\mathrm{of}\;\mathrm{PBC}\;\mathrm{in}\;\mathrm{NPs}\;}{\mathrm{Weight}\;\mathrm{of}\;\mathrm{NPs}}\times 100\%.$$

### 2.4. Stability Study

The freshly prepared drug loaded NPs, PBC@NPs, in ddH_2_O were stored at 4 °C, and stability samples were collected on days 0, 7, 14, 21, and 28 after storage. The particle size and zeta potential of each sample were measured using a zetasizer (Nano-ZS 90 Zetasizer, Malvern Instruments, Worcestershire, UK). Sucrose was selected as a lyoprotectant, and the freshly prepared PBC@NPs were homogeneously mixed with an equal volume of sucrose solution (5% *w*/*v*). The mixture was frozen at −80 °C and then freeze-dried overnight. The lyophilized samples were stored at −20 °C, and stability samples were collected at the same time intervals as previously mentioned. Each sample was resuspended in ddH_2_O and measured using a zetasizer.

### 2.5. In Vitro Release Study

To simulate the release of PBC in physiological and late endosomal environments, a phosphate-buffered solution (PBS, pH 7.4) and an acetic-buffered solution (pH 5.5) were used as the release media [39]. PBC@NPs were suspended in the release media in each vial covered with dialysis membrane (MWCO 12,000–14,000 Da). Each vial was placed into the tube containing a large quantity of the same release medium in a shaking bath under 100 rpm at 37 °C. At specific time intervals, the release medium was withdrawn and replaced with the same volume of fresh medium. The samples were then centrifuged at 30,000× *g* for 5 min at 4 °C, and the amount of PBC released was measured using HPLC. The release profiles were fitted by kinetic models, and the most appropriate release model with the highest correlation coefficient was selected. The time for 50% of drug release (*t_50_*) from PBC@NPs was then determined [40].

### 2.6. Cellular Uptake in a Co-Cultured BBB Model

A co-culture cell model consisting of bEnd.3 and U87-MG cells was established to assess the BBB penetration and glioma uptake capabilities of FITC labeled peptide-free PP_5k_ NPs and T7-modified PPT_5k_ NPs [41]. The bEnd.3 cells were seeded onto the upper chamber of a 6-insert cell (0.4 mm pore size, 24 mm diameter, SPL, Pochon, Republic of Korea) at a density of 2×10^5^ cells/well in DMEM, while U87-MG cells were seeded into the lower chamber using the same medium. NPs at concentrations of 0.15, 0.75, and 1.5 mg/mL in DMEM were added to the upper chambers of the Transwel^®^ supports and incubated at 37 °C for 4 and 12 h, respectively. After incubation, the medium was discarded and the cells were washed three times with cold PBS and then trypsinized. The cells were collected by centrifugation at 1300 rpm for 5 min and resuspended in PBS for analysis. The flow cytometer (Becton Dickinson, Franklin Lakes, NJ, USA) was used to measure the fluorescence intensity of cells at FL1 channel. A total of 10,000 events were analyzed for each sample, and the mean fluorescence intensity (MFI) was used to evaluate the cellular uptake of NPs in bEnd.3 and U87-MG cells.

### 2.7. In Vitro Transport of PBC@NPs in a bEnd.3 Monolayer 

The transport of PBC solution and PBC@NPs, including PBC@PP_5k_/PP_2k_ NPs, PBC@PPT_5k_/PP_2k_ NPs, and PBC@PPT_5k_/PPR_2k_ NPs, across the BBB was evaluated using a bEnd.3 monolayer. The PBC solution and PBC@NPs in DMEM at a PBC concentration of 10 μg/mL were added to the upper well of a 6-insert cell seeded with 2 × 10^5^ bEnd.3 cells per well. At specific time intervals, samples were collected from the lower chamber and replaced with the same volume of fresh medium. The collected samples were then centrifuged at 30,000× *g* 10,000 rpm for 5 min at 4 °C, and the concentrations of PBC were determined using HPLC. The transport efficiency of PBC was calculated by dividing the amount of PBC that crossed the bEnd.3 monolayer at each specific time interval by the initial amount of PBC that was added.

### 2.8. Cytotoxicity

The cytotoxicity of PBC solution and PBC@NPs in bEnd.3 and U87-MG cells was investigated using the MTT assay. The cells were uniformly seeded in 96-well plates at a density of 1 × 10^4^ cells/well in DMEM containing 10% FBS. After 24 h of incubation, the medium was removed, and PBC solution and PBC@NPs at various concentrations of PBC (0.5–52.4 nM) were added and incubated in 5% CO_2_ at 37 °C for 24 h. The MTT solution was then added to each well and continuously incubated for an additional 4 h. The supernatant was removed, and 100 μL of dimethyl sulfoxide (DMSO) was added to dissolve the formazan crystals. The absorbance was measured at 570 nm and 690 nm using a microplate reader (SpectraMax Paradigm, Molecular Devices, San Jose, CA, USA), and the cell viability (%) was calculated using equation 3. The half-maximal inhibitory concentration (IC_50_) was determined from the semi-log plot of cell viability (%) versus drug concentration.

(3) $$\mathrm{Cell}\;\mathrm{viability}\;(\%)=\frac{{\left[{\mathrm{OD}}_{570\mathrm{nm}}{-\mathrm{OD}}_{690\mathrm{nm}}\right]}_{\mathrm{sample}}}{{\left[{\mathrm{OD}}_{570\mathrm{nm}}{-\mathrm{OD}}_{690\mathrm{nm}}\right]}_{\mathrm{control}}}\times 100\%$$

### 2.9. Statistics

All data were presented as mean ± SD. All the statistics analysis was conducted by SigmaPlot 12.5 (Systat Software Inc., CA, USA). One-way ANOVA was for multiple-group analysis, and unpaired Student’s *t*-test was for two-group comparison. The statistical significance was defined as *p* < 0.05.

## 3. Results and Discussion

### 3.1. Characterization of Peptide-Conjugated PLGA-PEG Copolymers 

**Figure 1:**
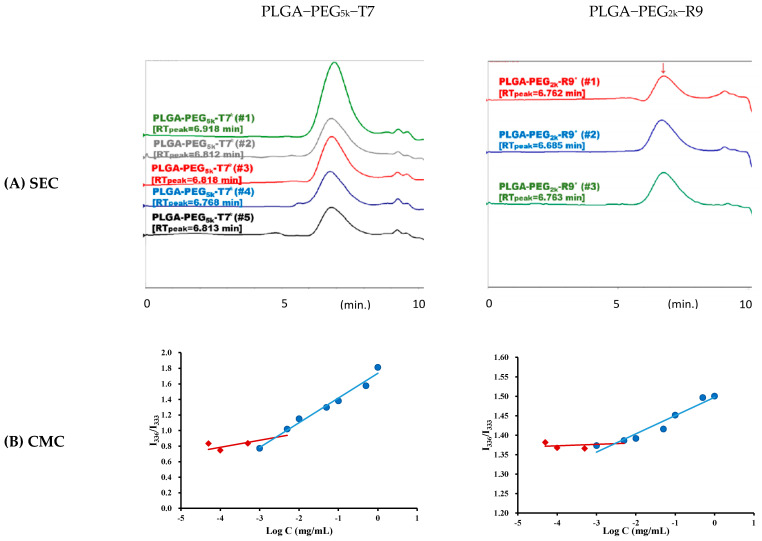
(**A**) SEC chromatograms of PLGA-PEG_5k_-T7 (n = 5) and PLGA-PEG_2k_-R9 (n = 3) including the retention time. (**B**) CMC plots of PLGA-PEG_5k_-T7 and PLGA-PEG_2k_-R9 using pyrene as a fluorescence probe. The emission wavelength was 390 nm, and the excitation fluorescence was recorded at 336 nm and 333 nm. The concentration at the intersection of the steep and the horizontal parts of the curve corresponds to the CMC value.

The peptide-conjugated PLGA-PEG_5k_-T7 and PLGA-PEG_2k_-R9 copolymers were synthesized, and their molecular weights and CMC values were determined (Figure 1). The calibration curve was established using polystyrene as the standards (MW 770–114,200 Da). The semilogarithmic plot of molecular weight and the corresponding retention time was constructed with R^2^ of 0.9955 which was applied to measure the number average molecular weight (Mn), weight average molecular weight (Mw), and polydispersity (PD) of polymers. The Mn, Mw and PD of PLGA-PEG_5k_-T7 were 28,000 ± 2500 g mol^−1^, 49,600 ± 2600 g mol^−1^, and 1.77 ± 0.11, respectively. Similarly, the Mw, Mn, and PD values of PLGA-PEG_2k_-R9 copolymer were 56,000 ± 5000 g mol^−1^, 32,000 ± 2600 g mol^−1^, and 1.77 ± 0.21, respectively. The CMC value, which represents the concentration at which micelle formation occurs in aqueous solution, was determined to be 2.53 × 10^−3^ mg/mL for PLGA-PEG_5k_-T7 and 2.83 × 10^−3^ mg/mL for PLGA-PEG_2k_-R9 based on the plots of fluorescence intensity ratio of I_336_/I_333_ versus logarithmic concentrations of peptide-conjugated copolymers.

### 3.2. Characterization of PBC Loaded Nanoparticles (PBC@NPs)

**Figure 2:**
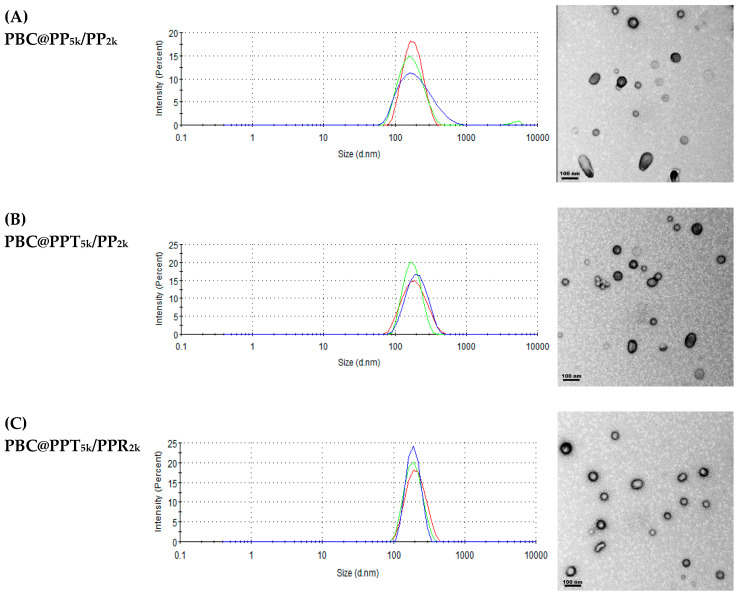
Particle size histograms (n = 3) and TEM images (scale bar: 100 nm, 100,000× magnification) of (**A**) PBC@PP_5k_/PP_2k_ NPs, (**B**) PBC@PPT_5k_/PP_2k_ NPs and (**C**) PBC@PPT_5k_/PPR_2k_ NPs.

According to Table 1, the yields of PBC@NPs were around 65%. The particle size of peptide-free PBC@PP_5k_/PP_2k_ NPs was 168.4 ± 4.3 nm, which slightly increased to 173.2 ± 7.8 nm when modified with T7-peptide (PBC@PPT_5k_/PP_2k_ NPs). The particle size of PBC@PPT_5k_/PPR_2k_ NPs, which were modified with T7/R9 dual-peptide, was further enlarged to 185.8 ± 4.4 nm. All PBC@NPs had a PDI < 0.2, indicating their monodisperse nature (Figure 2). Additionally, the PBC@NPs possessed negative zeta potential ranging from −17.8 ± 1.4 mV to −14.3 ± 1.0 mV. The PBC@PPT_5k_/PPR_2k_ NPs exhibit the least negative charge due to the presence of positively charged R9-peptide. The encapsulation efficiency and drug loading of PBC in PBC@NPs were similar regardless of the presence of peptides. TEM images of PBC@NPs were presented in Figure 2. Most of the NPs appeared individually dispersed with a spherical shape, consistent with the monodisperse nature of PBC@NPs.

### 3.3. Stability of PBC@NPs

**Figure 3:**
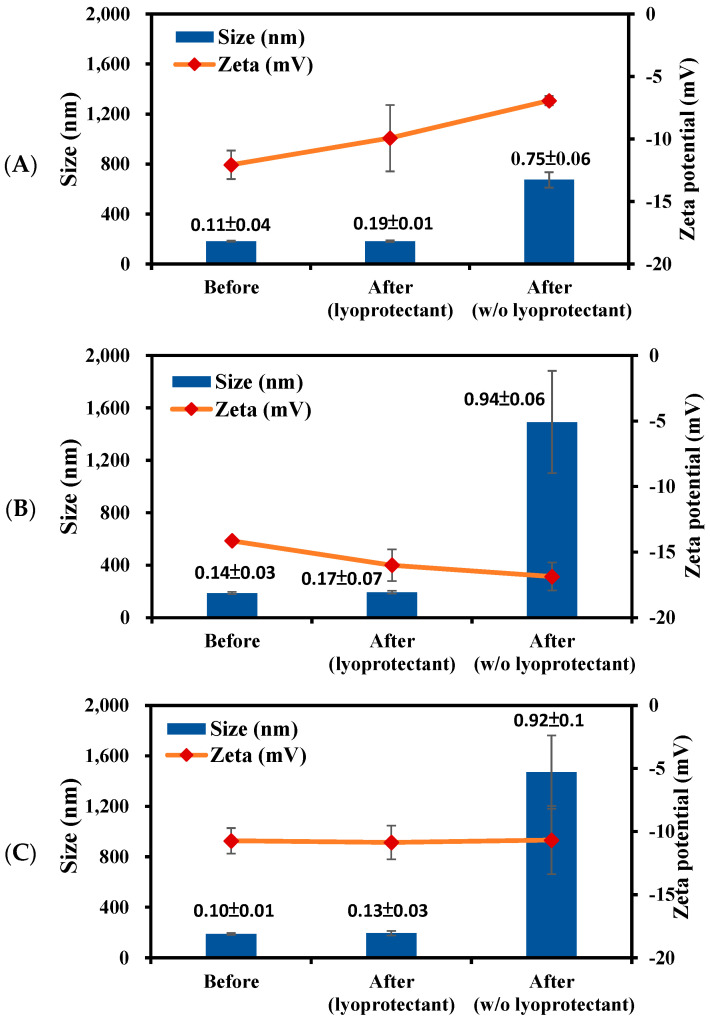
Lyoprotective effect of sucrose solution (5% *w*/*v*) on the particle size (bar), zeta potential (line), and PDI (indicated value) of (**A**) PBC@PP_5k_/PP_2k_ NPs, (**B**) PBC@PPT_5k_/PP_2k_ NPs, and (**C**) PBC@PPT_5k_/PPR_2k_ NPs. (n = 3, mean ± SD).

The stability of PBC@NPs in the absence or presence of sucrose as a lyoprotectant during lyophilization process was investigated, and the results are shown in Figure 3. Without lyoprotectant, PBC@NPs exhibited extreme changes in particle size, PDI, and zeta potential, resulting in severe aggregation. In contrast, the lyophilization of PBC@NPs in combination with sucrose were able to be fully redispersed, and their particle size, PDI, and zeta potential were preserved. It is likely that the sucrose molecules interact with the NPs surface via hydrogen bonding to prevent NPs collapse during lyophilization [42].

The particle size and zeta potential of PBC@NPs were maintained in ddH_2_O at 4 °C for 28 days, and the final to initial size ratio (S_f_/S_i_) was ranged from 0.9 to 1.1 which close to 1. This result indicated that the initial size was maintained and the NPs were considered stable under the storage condition [43,44]. Similarly, the lyophilized PBC@NPs preserved their original particle performance at −20 °C for 28 days, with Sf/Si ratios within 0.8–1.2, and without prominent change of zeta potentials.

### 3.4. In Vitro Release Study

**Figure 4:**
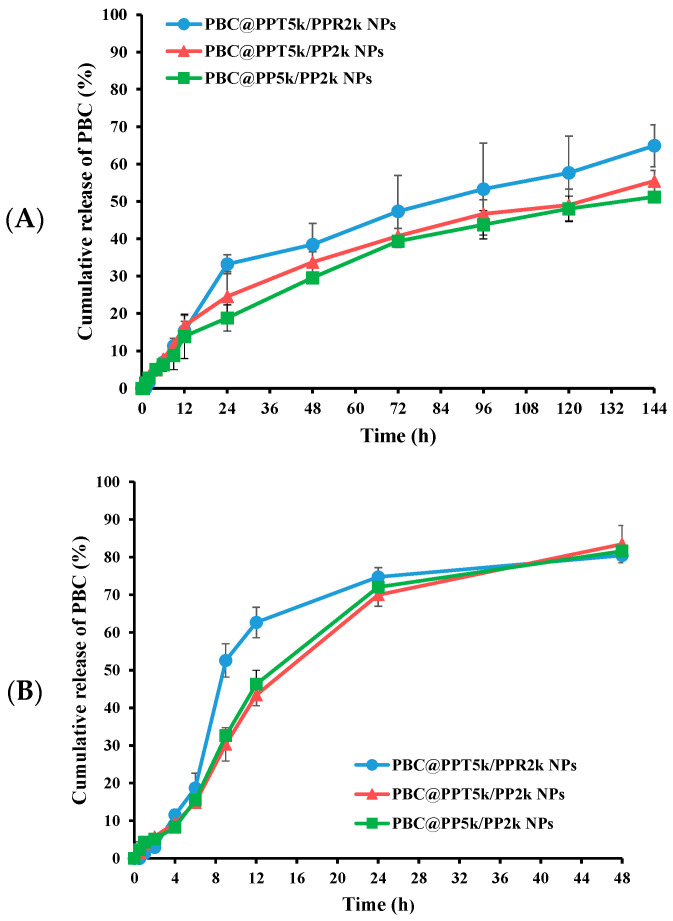
Cumulative release of PBC from PBC@NPs in (**A**) pH 7.4 and (**B**) pH 5.5 release media. (n = 3, mean ± SD).

The release of PBC from PBC@NPs was examined in simulated late endosome pH 5.5 and physiological pH 7.4, as shown in Figure 4. The release of PBC was found to be pH-dependent, with a faster release rate at pH 5.5 compared to pH 7.4. The initial burst release in the first 12 h followed by sustained release of drug was observed in both pH media. At pH 7.4, 13.9% ± 5.9–16.7% ± 2.8% of PBC was released from PBC@NPs, whereas at pH 5.5, 43.4% ± 2.8–62.7 ± 4.0% of PBC was released during the first 12 h. The hydrolysis of PLGA-PEG copolymer in the acidic environment further accelerated drug release, resulting in approximately 80% of PBC release in pH 5.5 at 48 h.

The release mechanism of PBC@NPs was investigated [45], and the results show that PBC@NPs exhibited diffusion-dominated release at pH 7.4, with *t_50_* ranging from 93.0 ± 12.0 h to 114.6 ± 13.9 h, depending on the type of polymers. However, at pH 5.5, PBC@NPs demonstrated a concentration-dependent release mechanism, with *t_50_* ranging from 11.5 ± 1.6 h to 18.0 ± 0.8 h. These findings indicate that the release of PBC from PBC@NPs was pH-dependent, and the NPs remained stable at normal physiological conditions but facilitated drug release in acidic microenvironments, such as endocytic and tumor sites [46].

### 3.5. Cellular Uptake in a Co-Cultured BBB Model

**Figure 5:**
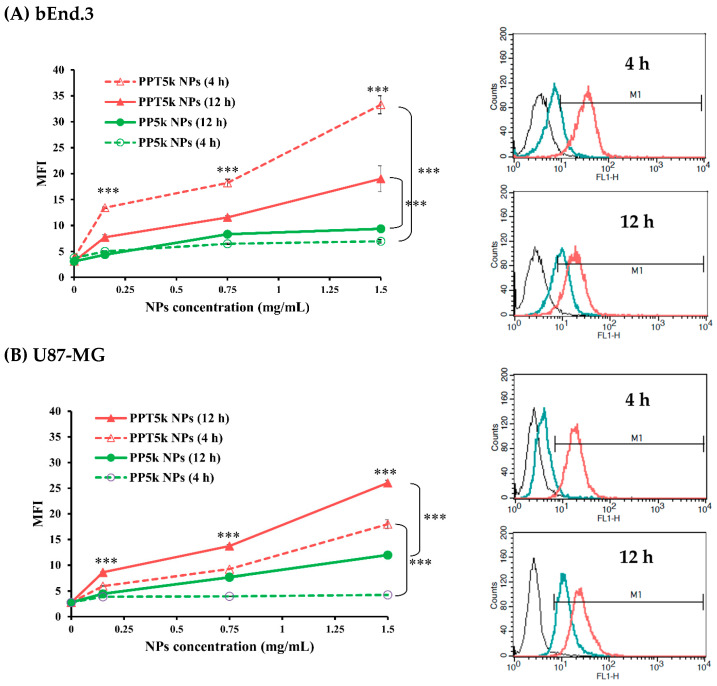
Cellular uptake of PP_5k_ NPs and PPT_5k_ NPs at different concentrations (0.15, 0.75, 1.5 mg/mL) in (**A**) bEnd.3 and (**B**) U87-MG cells for 4 h and 12 h in an in vitro co-cultured BBB model composed of U87-MG cells in the lower chamber and bEnd.3 monolayer in the upper chamber of a Transwell^®^ support (n = 3, mean ± SD, *** *p* < 0.001, compared to PP_5k_ NPs group). The flow cytometric plots (from left to right) represent untreated (black), PP_5k_ NPs (green) and PPT_5k_ NPs (red) treated at concentration of 1.5 mg/mL.

The potential of T7 peptide as a cellular uptake enhancer was evaluated in a co-cultured BBB model, which included a bEnd.3 monolayer in the upper chamber and U87-MG cells in the lower chamber of a Transwell^®^ support. The cellular uptake of FITC labeled T7 peptide-conjugated PPT_5k_ NPs and peptide-free PP_5k_ NPs was assessed in both bEnd.3 and U87-MG cells using flow cytometry. Figure 5 indicates that PPT_5k_ NPs exhibited a dose-dependent cellular uptake, which significantly increased in both bEnd.3 and U87-MG cells after 4 h of incubation compared to peptide-free PP_5k_ NPs. When the incubation time was extended from 4 h to 12 h, the cellular uptake of PPT_5k_ NPs in U87-MG cells was further enhanced. However, less PPT_5k_ NPs were observed in bEnd.3 cells after 12 h of incubation compared to 4 h of incubation, indicating successful penetration of PPT_5k_ NPs across the bEnd.3 monolayer and into U87-MG tumor cells. In contrast, the cellular uptake of peptide-free PP_5k_ NPs was limited in both bEnd.3 and U87-MG cells regardless of incubation time. These results indicated that T7 peptide effectively enhances the cellular uptake of PPT_5k_ NPs across the BBB and into the glioma cancer cells.

### 3.6. Transport of PBC@NPs across bEnd.3 Cell Model

**Figure 6:**
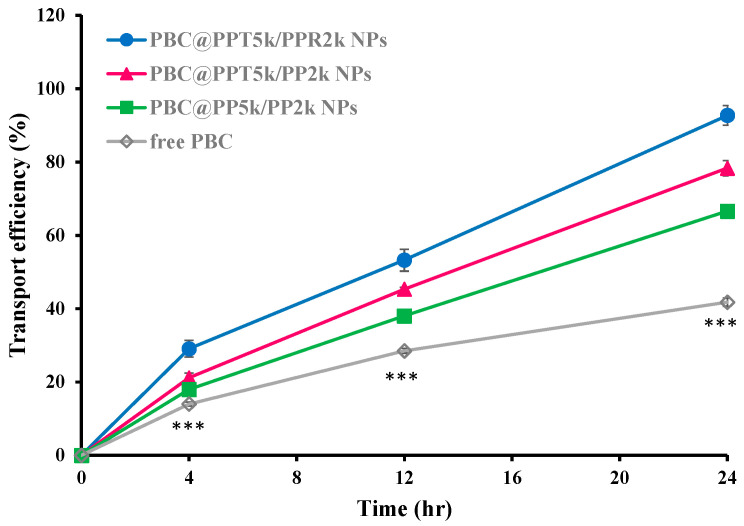
In vitro transport of PBC@NPs (PBC@PP_5k/_PP_2k_ NPs, PBC@PPT_5k_/PP_2k_ NPs, and PBC@PPT_5k_/PPR_2k_ NPs) and PBC solution across the bEnd.3 monolayer. (n = 3, mean ± SD, *** *p* < 0.001: free PBC compared to PBC@NPs).

Figure 6 illustrates the transport of drug-loaded PBC@NPs, including PBC@PP_5k_/PP_2k_ NPs, PBC@PPT_5k_/PP_2k_ NPs, and PBC@PPT_5k_/PPR_2k_ NPs, across the bEnd.3 monolayer for 24 h. The result shows the transport of the drug across the bEnd.3 monolayer at the end of 24 h in the following order of PBC@PPT_5k_/PPR_2k_ NPs (92.7 ± 2.7%) > PBC@PPT_5k_/PP_2k_ NPs (78.3 ± 2.1%) > PBC@PP_5k_/PP_2k_ NPs (66.6 ± 1.6%) > PBC solution (41.8 ± 11.1%). It is widely acknowledged that the BBB layer represents a critical barrier for drug delivery to the brain and contains various efflux transporters on the brain endothelial cells. Our results confirm that the delivery of PBC across the brain barrier can be facilitated by NPs, especially the dual-peptide modified PBC@PPT_5k_/PPR_2k_ NPs.

### 3.7. Cytotoxicity of PBC@NPs in U87-MG Cells

**Figure 7:**
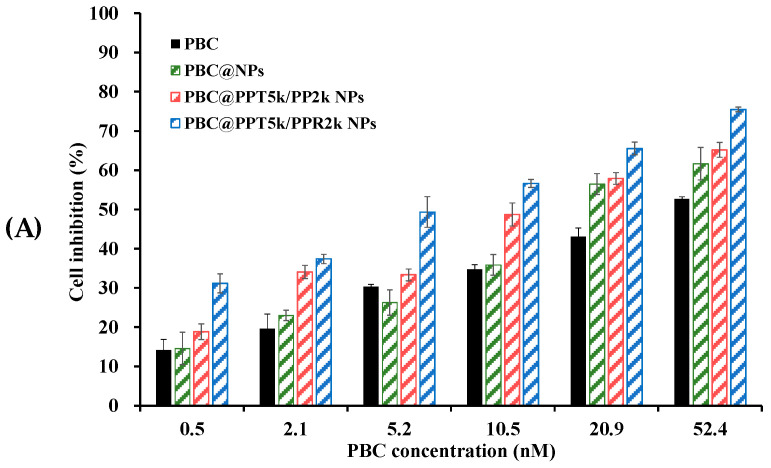

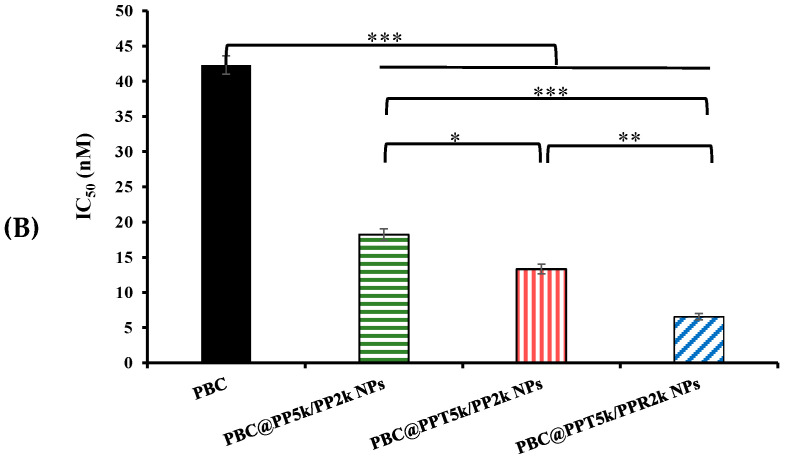
(**A**) The U87-MG cell inhibition after treatment of PBC solution and PBC@NPs at drug concentrations of 0.5–52.4 nM for 24 h. (**B**) The IC_50_ of free PBC, PBC@PP_5k_/PP_2k_ NPs, PBC@PPT_5k_/PP_2k_ NPs, and PBC@PPT_5k_/PPR_2k_ NPs. (n = 3, mean ± SD). * *p* < 0.05, ** *p* < 0.01 and *** *p* < 0.001.

Figure 7 shows the cytotoxicity of PBC@NPs and PBC solution in U87-MG cancer cells after 24 h of treatment. The results indicate that both PBC@NPs and PBC solution inhibited cell proliferation in a dose-dependent manner. Among the PBC@NPs, the T7-modified PBC@PPT_5k_/PP_2k_ NPs exhibited a stronger inhibitory effect than the peptide-free PBC@PP_5k_/PP_2k_ NPs. Furthermore, the T7/R9 dual-peptide functionalized PBC@PPT_5k_/PPR_2k_ NPs exhibited a greater reduction in cell viability compared to the T7-peptide modified PBC@PPT_5k_/PP_2k_ NPs. The IC_50_ values for free PBC and PBC@NPs are in the following order of free PBC > PBC@PP_5k_/PP_2k_ NPs > PBC@PPT_5k_/PP_2k_ NPs > PBC@PPT_5k_/PPR_2k_ NPs. The use of PBC@NPs resulted in a significant increase in apoptosis of U87-MG glioma cells, with a 2.3~6.5-fold increment compared to free PBC. The dual peptide modified PBC@PPT_5k_/PPR_2k_ NPs exhibited the most cytotoxic response. These findings suggest that PBC@NPs are capable of effectively evading efflux transporters on the surface of cancer cells, following their internalization into tumor sites through the enhanced permeability and retention (EPR) effect [47,48]. The T7-peptide initiates receptor-mediated endocytosis of PBC@PPT_5k_/PP_2k_ NPs through TfR on the U87-MG cell surface, which further allows cell penetration by the R9-peptide on the NPs. The electrostatic interaction between the R9-peptide and cancer cells results in the promotion of cell apoptosis by PBC@PPT_5k_/PPR_2k_ NPs.

## 4. Conclusions

The dual-peptide functionalized NPs, comprising of a specific targeting ligand T7-peptide and a non-specific cell-penetrating R9-peptide, have been successfully developed for palbociclib in glioma therapy. The PBC@NPs have negative zeta potential and particle size < 200 nm with narrow size distribution. The freshly prepared PBC@NPs in ddH_2_O at 4 °C and the lyophilized PBC@NPs at −20 °C were able to maintain particle performance at least for 28 days. The release of PBC from PBC@NPs exhibited pH-dependent character where a faster release was observed at pH 5.5 (a simulated late endosome environment) as compared to physiological pH 7.4. The dual-peptide-functionalized NPs significantly enhanced PBC transport across bEnd.3 and efficiently inhibition of U87-MG cell growth, as compared to PBC alone. The corresponding IC_50_ values were in the order of free PBC > PBC@PP_5k_/PP_2k_ NPs > PBC@PPT_5k_/PP_2k_ NPs > PBC@PPT_5k_/PPR_2k_ NPs. The beneficial cytotoxicity of PBC delivered by dual-peptide-functionalized NPs for treatment of brain glioma was elucidated.

## Figures and Tables

**Table 1:** Characteristics of PBC@NPs. (n = 3, mean ± SD).

NPs	Yield (%)	Size (nm)	PdI	Zeta (mV)	EE (%)	DL (%)
PBC@PP_5k_/PP_2k_ NPs	65.1 ± 2.5	168.4 ± 4.3	0.15 ± 0.04	−16.0 ± 1.4	60.6 ± 4.4	5.3 ± 0.4
PBC@PPT_5k_/PP_2k_ NPs	65.7 ± 6.4	173.2 ± 7.8	0.13 ± 0.03	−17.8 ± 1.4	62.0 ± 5.3	5.3 ± 0.1
PBC@PPT_5k_/PPR_2k_ NPs	68.9 ± 2.5	185.8 ± 4.4	0.09 ± 0.07	−14.3 ± 1.0	60.5 ± 3.6	5.0 ± 0.1

## Data Availability

No new data were available.

## Abbreviations

**Abbreviation**	
PBC	Palbociclib
PBC@NP	Palbociclib loaded NPs
PP_5k_	PLGA-PEG_5k_ copolymer (MW of PEG ~5000 Da)
PP_2k_	PLGA-PEG_2k_ copolymer (MW of PEG ~2000 Da)
PPT_5k_	T7 peptide conjugated with PP_5k_
PPR_2k_	R9 peptide conjugated with PP_2k_
PP_5k_/PP_2k_ NP (peptide-free NPs)	NPs prepared by PP_5k_ and PP_2k_ (3:1 *w*/*w*)
PPT_5k_/PP_2k_ NPs (T7 peptide-conjugated NPs)	NPs prepared by PPT_5k_ and PP_2k_ (3:1 *w*/*w*)
PPT_5k_/PPR_2k_ NPs (T7/R9 dual peptide conjugated NPs)	NPs prepared by PPT_5k_ and PPR_2k_ (3:1 *w*/*w*)

## References

[1] Mair M.J., Geurts M., van den Bent M.J., Berghoff A.S. (2021). A basic review on systemic treatment options in WHO grade II–III gliomas. Cancer Treat. Rev..

[2] Shah F.H., Salman S., Idrees J., Idrees F., Shah S.T.A., Khan A.A., Ahmad B. (2020). Current progress of phytomedicine in glioblastoma therapy. Curr. Med. Sci..

[3] Erthal L.C.S., Gobbo O.L., Ruiz-Hernandez E. (2021). Biocompatible copolymer formulations to treat glioblastoma multiforme. Acta Biomater..

[4] Turner N.C., Ro J., Andr F., Loi S., Verma S., Iwata H., Harbeck N., Loibl S., Bartlett C.H., Zhang K. (2015). Palbociclib in hormone-receptor-positive advanced breast cancer. N. Engl. J. Med..

[5] Asghar U., Witkiewicz A.K., Turner N.C., Knudsen E.S. (2015). The history and future of targeting cyclin-dependent kinases in cancer therapy. Nat. Rev. Drug Discov..

[6] Spring L., Bardia A., Modi S. (2016). Targeting the cyclin D-cyclin-dependent kinase (CDK)4/6-retinoblastoma pathway with selective CDK 4/6 inhibitors in hormone receptor-positive breast cancer: Rationale, current status, and future directions. Discov. Med..

[7] Michaud K., Solomon D.A., Oermann E., Kim J.S., Zhong W.Z., Prados M.D., Ozawa T., James C.D., Waldman T. (2010). Pharmacologic inhibition of cyclin-dependent kinases 4 and 6 arrests the growth of glioblastoma multiforme intracranial xenografts. Cancer Res..

[8] Schroder L.B., McDonald K.L. (2015). CDK4/6 Inhibitor PD0332991 in glioblastoma treatment: Does it have a future?. Front. Oncol..

[9] Barton K.L., Misuraca K., Cordero F., Dobrikova E., Min H.D., Gromeier M., Kirsch D.G., Becher O.J. (2013). PD-0332991, a CDK4/6 inhibitor, significantly prolongs survival in a genetically engineered mouse model of brainstem glioma. PLoS ONE.

[10] de Gooijer M.C., Zhang P., Thota N., Mayayo-Peralta I., Buil L.C.M., Beijnen J.H., van Tellingen O. (2015). P-glycoprotein and breast cancer resistance protein restrict the brain penetration of the CDK4/6 inhibitor Palbociclib. Investig. New Drugs.

[11] Bronner S.M., Merrick K.A., Murray J., Salphati L., Moffat J.G., Pang J., Sneeringer C.J., Dompe N., Cyr P., Purkey H. (2019). Design of a brain-penetrant CDK4/6 inhibitor for glioblastoma. Bioorg. Med. Chem. Lett..

[12] Oberoi R.K., Parrish K.E., Sio T.T., Mittapalli R.K., Elmquist W.F., Sarkaria J.N. (2016). Strategies to improve delivery of anticancer drugs across the blood-brain barrier to treat glioblastoma. Neuro. Oncol..

[13] Mehata A.K., Singh V., Vikas S.N., Mandal A., Dash D., Koch B., Muthu M.S. (2023). Chitosan-g-estrone nanoparticles of palbociclib vanished hypoxic breast tumor after targeted delivery: Development and ultrasound/photoacoustic imaging. ACS Appl. Mater. Interfaces.

[14] Persha H.E., Kato S., De P., Adashek J.J., Sicklick J.K., Subbiah V., Kurzrock R. (2022). Osteosarcoma with cell-cycle and fibroblast growth factor genomic alterations: Case report of molecular tumor board combination strategy resulting in long-term exceptional response. J. Hematol. Oncol..

[15] Oh S., Kim B.J., Singh N.P., Lai H., Sasaki T. (2009). Synthesis and anti-cancer activity of covalent conjugates of artemisinin and a transferrin-receptor targeting peptide. Cancer Lett..

[16] Bi Y., Liu L., Lu Y., Sun T., Shen C., Chen X., Chen Q., An S., He X., Ruan C. (2016). T7 peptide-functionalized PEG-PLGA micelles loaded with carmustine for targeting therapy of glioma. ACS Appl. Mater. Interfaces.

[17] Cui Y., Zhang M., Zeng F., Jin H., Xu Q., Huang Y. (2016). Dual-targeting magnetic PLGA nanoparticles for codelivery of paclitaxel and curcumin for brain tumor therapy. ACS Appl Mater. Interfaces.

[18] Han L., Huang R., Liu S., Huang S., Jiang C. (2010). Peptide-conjugated PAMAM for targeted doxorubicin delivery to transferrin receptor overexpressed tumors. Mol. Pharm..

[19] Lee J.H., Engler J.A., Collawn J.F., Moore B.A. (2001). Receptor mediated uptake of peptides that bind the human transferrin receptor. Eur. J. Biochem..

[20] Wang X., Mao W., Wang Z., Li X., Xiong Y., Lu H., Wang X., Yin H., Cao X., Xin H. (2020). Enhanced anti-brain metastasis from non-small cell lung cancer of osimertinib and doxorubicin co-delivery targeted nanocarrier. Inter. J. Nanomed..

[21] Wang H., Chen W., Wu G., Kong J., Yuan S., Chen L. (2021). A magnetic T7 peptide&AS1411 aptamer-modified microemulsion for triple glioma-targeted delivery of shikonin and docetaxel. J. Pharm. Sci..

[22] Li C., Guan N., Liu F. (2023). T7 peptide-decorated exosome-based nanocarrier system for delivery of Galectin-9 siRNA to stimulate macrophage repolarization in glioblastoma. J. Neurooncol..

[23] Tang L., Zhang R., Wang Y., Zhang X., Yang Y., Zhao B., Yang L. (2023). A simple self-assembly nanomicelle based on brain tumor-targeting peptide-mediated siRNA delivery for glioma immunotherapy via intranasal administration. Acta Biomater..

[24] Stiltner J., McCandless K., Zahid M. (2021). Cell-penetrating peptides: Applications in tumor diagnosis and therapeutics. Pharmaceutics.

[25] Chiarpotti M.V., Longo G.S., Del Pópolo M.G. (2021). Nanoparticles modified with cell penetrating peptides: Assessing adsorption on membranes containing acidic lipids. Colloids Surf. B Biointerfaces.

[26] McErlean E.M., Ziminska M., McCrudden C.M., McBride J.W., Loughran S.P., Cole G., Mulholland E.J., Kett V., Buckley N.E., Robson T. (2021). Rational design and characterisation of a linear cell penetrating peptide for non-viral gene delivery. J. Control. Release.

[27] Sadeghian I., Heidari R., Raee M.J., Negahdaripour M. (2022). Cell-penetrating peptide-mediated delivery of therapeutic peptides/proteins to manage the diseases involving oxidative stress, inflammatory response and apoptosis. J. Pharm. Pharmacol..

[28] Kurrikoff K., Vunk B., Langel Ü. (2021). Status update in the use of cell-penetrating peptides for the delivery of macromolecular therapeutics. Expert Opin. Biol. Ther..

[29] Falato L., Gestin M., Langel Ü. (2021). Cell-penetrating peptides delivering siRNAs: An Overview. Methods Mol. Biol..

[30] Zhang C., Yuan W., Wu Y., Wan X., Gong Y. (2021). Co-delivery of EGFR and BRD4 siRNA by cell-penetrating peptides-modified redox-responsive complex in triple negative breast cancer cells. Life Sci..

[31] Feldman K.S., Pavlou M.P., Zahid M. (2021). Cardiac targeting peptide: From identification to validation to mechanism of transduction. Methods Mol. Biol..

[32] Ghorai S.M., Deep A., Magoo D., Gupta C., Gupta N. (2023). Cell-penetrating and targeted peptides delivery systems as potential pharmaceutical carriers for enhanced delivery across the blood-brain barrier (BBB). Pharmaceutics.

[33] Danhier F., Ansorena E., Silva J.M., Coco R., Breton A.L., Préat V. (2012). PLGA-based nanoparticles: An overview of biomedical applications. J. Control. Release.

[34] Wicki A., Witzigmann D., Balasubramanian V., Huwyler J. (2015). Nanomedicine in cancer therapy: Challenges, opportunities, and clinical applications. J. Control. Release.

[35] Lin W.J., Kao L.T. (2014). Cytotoxic enhancement of hexapeptide-conjugated micelles in EGFR high-expressed cancer cells. Expert Opin. Drug Deliv..

[36] Huang H.L., Lin W.J. (2020). Dual peptide-modified nanoparticles improve combination chemotherapy of etoposide and siPIK3CA against drug-resistant small cell lung carcinoma. Pharmaceutics.

[37] He G.Z., Lin W.J. (2021). Peptide-functionalized nanoparticles-encapsulated cyclin-dependent kinases inhibitor seliciclib in transferrin receptor overexpressed cancer cells. Nanomaterial.

[38] Wu R., Tian M., Shu C., Zhou C., Guan W. (2022). Determination of the critical micelle concentration of surfactants using fluorescence strategies. Soft Matter.

[39] O’Leary B., Finn R.S., Turner N.C. (2016). Treating cancer with selective CDK4/6 inhibitors. Nat. Rev. Clin. Oncol..

[40] Alai M., Lin W.J. (2013). A novel once daily microparticulate dosage form comprising lansoprazole to prevent nocturnal acid breakthrough in the case of gastro-esophageal reflux disease: Preparation, pharmacokinetic and pharmacodynamic evaluation. J. Microencapsul..

[41] Biemans E., Jakel L., de Waal R.M.W., Kuiperij H.B., Verbeek M.M. (2017). Limitations of the hCMEC/D3 cell line as a model for Abeta clearance by the human blood-brain barrier. J. Neurosci. Res..

[42] Goyal R., Macri L., Kohn J. (2015). Formulation strategy for the delivery of cyclosporine a: Comparison of two polymeric nanospheres. Sci. Rep..

[43] Dorati R., DeTrizio A., Spalla M., Migliavacca R., Pagani L., Pisani S., Chiesa E., Conti B., Modena T., Genta I. (2018). Gentamicin sulfate PEG-PLGA/PLGA-H nanoparticles: Screening design and antimicrobial effect evaluation toward clinic bacterial isolates. Nanomaterials.

[44] Li Y., Monteiro-Riviere N.A. (2016). Mechanisms of cell uptake, inflammatory potential and protein corona effects with gold nanoparticles. Nanomedicine.

[45] Dash S., Murthy P.N., Nath L., Chowdhury P. (2010). Kinetic modeling on drug release from controlled drug delivery systems. Acta Pol. Pharm..

[46] Du J., Lane L.A., Nie S. (2015). Stimuli-responsive nanoparticles for targeting the tumor microenvironment. J. Control. Release.

[47] Creixell M., Peppas N.A. (2012). Co-delivery of siRNA and therapeutic agents using nanocarriers to overcome cancer resistance. Nano Today.

[48] Kobayashi H., Watanabe R., Choyke P.L. (2014). Improving conventional enhanced permeability and retention (EPR) effects; what is the appropriate target?. Theranostics.

